# Age and sex-related differences in epidemiology, treatment, and mortality of patients with ST-segment elevation myocardial infarction in Iran

**DOI:** 10.34172/jcvtr.2023.32887

**Published:** 2023-12-30

**Authors:** Mehdi Darabi, Reza Heidari Moghaddam, Farzaneh Godarzi, Sahar Karami, Soraya Siabani, Nahid Salehi

**Affiliations:** ^1^Cardiovascular Research Center, Health Institute, Imam-Ali Hospital, Kermanshah University of Medical Sciences, Kermanshah, Iran; ^2^Department of Health Education and Health Promotion, Kermanshah University of Medical Sciences, Kermanshah, Iran

**Keywords:** Sex, Mortality, Treatment, STEMI, Risk factors

## Abstract

**Introduction:**

Few studies have investigated the characteristics of patients with ST elevation myocardial infarction (STEMI) according to age and sex in Iran. This study aims to investigate the risk factors profile, treatment, and mortality of STEMI based on age and sex.

**Methods:**

From 10th June 2016 to December 2019 a total of 2816, STEMI patients referred to the Imam Ali heart center of Kermanshah were included in the study. Profile of the risk factors, epidemiology, treatment and 30- day mortality for all cases in the age categories of 18-49, 50-64, and≥65 years were studied.

**Results:**

There were 1256 (44.6%) middle-aged STEMI patients, and 2181 (77.45%) were male. The elderly had a longer median door-to-balloon and symptom-to-balloon time and received less primary PCI. In the absence of primary PCI, the rate of 30-day mortality in women was higher than in men, and the mortality rate increased with age. The risk of death in middle-aged women was higher than that of men. Also, in the middle-aged group, after multivariable adjustment, previous bypass surgery, diabetes, and Killip class≥2 was associated with significant increase in the risk of death.

**Conclusion:**

The present study showed that young people with STEMI had a high risk of heart failure and anterior infarction compared to the older age groups. Women had more risk factors for STEMI and a higher mortality rate than men. Therefore, there is a need to educate young age groups and women to modify their lifestyles and intervene in the risk factors of heart diseases.

## Introduction

 Myocardial infarction (MI) is a major health concern worldwide, including in Iran.^1^ Approximately 15 million people worldwide experience MI each year. ST-elevation myocardial infarction (STEMI) is one of the most critical and life-threatening manifestations of cardiovascular disease, affecting all age groups. The 30-day mortality rate increases from 3% in patients younger than 65 years to 30% in patients older than 85 years.^2-4^ Although STEMI is relatively uncommon in young people, more than half of STEMI cases occur in people under 65 years of age, and approximately 10% of all STEMI cases occur in people under 45 years of age.^5-8^ Previous studies have shown significant differences in the risk factors and outcomes of STEMI in young patients compared to the elderly.^9,10^ Younger people probably have a history of smoking, illicit drug use, and oral contraceptive use, but they likely have fewer co-morbidities such as diabetes, high blood pressure, and coronary artery disease.^8,10-12^ Several studies have investigated differences in mortality between men and women in patients with STEMI. Young women exhibited a higher mortality rate than young men, while no difference was observed in the elderly.^13,14^ Enhanced understanding of the epidemiology and consequences of STEMI leads to insights into the etiology of the disease, risk factors, rational prioritization, allocation of treatment resources, and more appropriate planning within the health system. To the best of our knowledge, few studies have been conducted on the differences in epidemiology, treatment, and mortality hazard ratio concerning the age and sex of STEMI patients in Iran. Therefore, the purpose of this study was to evaluate the variations in the profile of risk factors, clinical characteristics, treatment patterns, and outcomes in young, middle-aged, and elderly individuals with STEMI based on sex.

## Materials and Methods

 This cohort study is part of the registry of patients with STEMI at the Imam Ali Cardiology Center of Kermanshah University of Medical Sciences. It is the main cardiovascular center in the west of Iran, which serves a population of about two million. The data are designed based on European Observational Research Program (EORP) standards.^15^ All patients aged 18 and older with STEMI, diagnosed by cardiologists according to recent guidelines and admitted between 2016 and 2019, were included in the present study.^16^ STEMI patients admitted to Imam Ali Cardiovascular Center more than 24 hours before referral and patients with cardiac arrest outside the hospital were excluded from the study. In this study, trained physicians and nurses collected demographic and clinical information, such as medical history, time of symptom onset, and transfer to the hospital, through personal interviews with patients and/or their attendants. The time between symptom onset and hospital admission was calculated. The history of cardiovascular events (previous MI, stroke, or chronic heart failure), cardiac interventions (PCI or coronary artery bypass graft surgery), biochemical findings, diabetes, and blood pressure were recorded based on self-report and confirmed diagnoses by healthcare professionals. Inclusion criteria were diagnosis of STEMI with chest pain or equivalent symptoms lasting more than 20 minutes in the 24 hours before admission and electrocardiographic changes compatible with elevations or left bundle branch block new or presumed new ST-segment according to the third definition of MI by the European Society of Cardiology/ACCF/AHA/ World Heart Federation Task Force for the Universal Definition of Myocardial Infarction.^17^ Two groups were considered based on blood pressure at the time of admission (SBP: < 140/ ≥ 140 mmHg, DBP: < 80/ ≥ 80 mmHg) or history or use of antihypertensive medications were considered. BMI was calculated by dividing weight (in kg) by the square of the height (in *m*^2^). Heart rate and lipid profile, including low-density lipoprotein cholesterol (LDL-C), with high LDL-C defined as > 160 mg/dL and low high-density lipoprotein cholesterol (HDL-C) as < 40 mg/dL in men and < 50 mg/dL in women, were also included. Additionally, blood sugar levels were measured on the first day of admission. Fasting blood sugar levels equal to or greater than 126 or history or use of antihyperglycemic medications are considered indicative of diabetes. Current smoking status is determined by the consumption of one or more cigarettes within the past 30 days. Door-to-balloon time is defined as the period from a patient’s arrival and triage in the hospital to the inflation of the balloon in non-transfer scenarios. This interval, also known as the Door-to-Balloon time, is measured from the arrival of a STEMI patient in the Emergency Department to the performance of pPCI in the cath lab. According to the American Heart Association, this interval should be less than 90 minutes.

 Percutaneous coronary intervention (PCI) was defined based on the National Cardiovascular Data Registry. The patients were between 18 and 93 years old and were divided into three age groups: 18-49, 50-64, and above 65. Baseline clinical and laboratory characteristics of the patients were compared among the three age groups, as well as sex in each age group. Continuous variables were expressed as mean (SD) and median (interquartile range [IQR]), and categorical variables as frequency and percentage. The comparison of continuous variables was done using the Mann–Whitney U test, and for categorical variables, the chi-square test or Fisher’s exact test was used. One-way ANOVA used for comparing differences between three age groups according to sex. The primary outcome was death within 30 days after STEMI. After admitting the patients, the trained nurses called the family members or companions by phone and collected information about the patient’s vital condition. Follow-up time was defined as the time to the investigated event (death) or being lost to follow-up, or 30 days after STEMI, whichever occurred earlier. The hazard ratio was calculated based on the Cox proportional hazards model and was used to compare the outcomes among different age groups. Adjustments were made for covariates associated with STEMI outcomes, such as BMI, blood pressure, diabetes, and dyslipidemia. All statistical analyses were performed using Stata SE (V.14.2) (Stata Corp LP; College Station, TX, USA) at a significance level of 0.05.

## Results

 Of the 2816 STEMI patients considered in this study, 45% were in the age group of 50-64. Table 1 shows baseline characteristics of the study population. More than three-quarters of the patients were men. The proportion of women with STEMI increases with age, while the highest incidence of STEMI in middle age is reported for men. A history of hypertension, diabetes, and dyslipidemia was common in the older population, but higher average LDL, triglyceride, BMI, heart rate, lower HDL, and a higher ratio of current smoking were reported for the younger population compared to middle-aged and older people. The mean Door-to-balloon time and Symptom-to-balloon time were significantly lower in the young and middle-aged groups than in the elderly. The proportion of patients with Killip class 2 and 3 increases with age, but in Killip class 4, the middle-aged have the highest frequency. There are differences between epidemiological and clinical risk factors among men and women in different age groups (Table 2). The results have shown that blood pressure, diabetes, and dyslipidemia were higher in women (in all three groups) than in men. Additionally, compared to men, a higher average BMI in middle-aged and elderly women and a higher heart rate in young and middle-aged women were reported (P < 0.05). Although a higher frequency of current smoking in all three age groups was reported for men compared to women. Also, a longer Door-to-balloon time was reported for middle-aged women, and a longer Symptom to balloon time was recorded for young and middle-aged women than for men of the same age. In both groups of women and men, BMI shows a significant difference in age groups.

**Table 1:** Baseline characteristics of the patients

**Variable**	**Young 18-49 (n=548)**	**Middle age 50-64 (n=1256)**	**Older≥65 (n=1012)**	***P*** value*
Gender Men Women	487 (88.87) 61 (11.13)	1044 (83.12) 212 (16.88)	650 (64.23) 362 (35.77)	< 0.001
Hypertension n (%) SBP Diabetes n (%) Dyslipidemia n (%) LDL mean (SD) HDL mean (SD) Triglyceride mean (SD)	222 (40.81) 131.34 (27.22) 81 (14.81) 106 (19.38) 108.41(32.42) 40.95 (9.26) 164.65 (109.81)	567 (45.22) 133.83 (30.03) 296 (23.59) 297 (23.67) 104.69 (30.69) 40.94 (8.86) 147.68 (100.55)	478 (47.37) 135.56 (32.18) 266 (26.23) 254 (25.32) 100.91 (30.58) 42.17 (9.55) 122.37 (66.51)	0.046 0.031 < 0.001 0.009 < 0.001 0.008 < 0.001
BMI, mean (SD)	27.41 ( ± 4.08)	26.36 ( ± 3.96)	25.45 ( ± 4.15)	< 0.001
Heart rate, mean (SD)	80.38 (20.37)	77.76 (19.07)	77.52 (21.95)	0.018
Door-to-balloon time (min) (median (IQR))	91 (74-120)	95 (75-122)	102.5 (82-137)	< 0.001
Symptom to balloon time (min) (median (IQR))	235 (165-387.5)	250 (170-385)	285 (205-435)	< 0.001
Previous MI, n (%)	48 (8.76)	161 (12.82)	127 (12.65)	0.030
Previous CABG, n (%)	12 (2.19)	39 (3.11)	41 (4.08)	0.200
Congestive Heart failure, n (%)	12 (2.19)	34 (2.72)	48 (4.77)	0.022
Previous PCI, n (%)	28 (5.11)	94 (7.49)	59 (5.88)	0.109
Previous strok, n (%)	7 (1.28)	55 (4.39)	99 (9.83)	< 0.001
Previous angina, n (%)	75 (13.69)	218 (17.38)	181 (18.01)	0.008
Cerebrovascular accident n (%)	2 (0.37)	4 (0.32)	5 (0.50)	0.303
Chest pain, n (%)	546 (99.64)	1250 (99.52)	995 (98.32)	0.595
Killip class n (%) Class 1 Class 2 Class 3 Class 4	502(91.61) 15 (2.74) 2 (0.36) 29 (5.29)	1124 (89.49) 59 (4.70) 4 (0.32) 69 (5.49)	880 (87.04) 61 (6.03) 19 (1.88) 51 (5.04)	< 0.001
Current Smoking n (%)	327 (59.67)	706 (56.21)	337 (33.30)	< 0.001

BMI, body mass index; CABG, coronary artery bypass graft; PCI, Percutaneous coronary intervention *P for comparing differences between three age groups. *P*<0.05 is statistically significant.

**Table 2:** Baseline Characteristics Sorted by Sex

**Variable**	**18-49 (n=548)**			**50-64 (n=1256)**			**≥65 (n=1012)**		P*	***P*** for age**
	**Women (61)**	**Men (487)**	P*	**Women (212)**	**Men (1044)**	P*	**Women (362)**	**Men (650)**		
Hypertension, n(%)	25 (40.0)	197 (40.45)	0.976	101 (47.64)	466 (44.72)	0.436	175 (48.48)	303 (46.76)	0.601	Women: 0.555 Men: 0.132
Diabetes, n (%)	25 (40.98)	56 (11.52)	< 0.001	94 (44.34)	202 (19.37)	< 0.001	136 (37.78)	130 (20.03)	< 0.001	Women: 0.301 Men: < 0.001
Dyslipidemia,n (%)	19 (31.15)	87 (17.90)	0.014	94 (44.34)	203 (19.46)	< 0.001	139 (38.94)	115 (29.70)	< 0.001	Women: 0.178 Men: 0.306
BMI, mean (SD)	27.91 (4.42)	27.35(4.04)	0.321	27.63 (4.61)	26.10 (3.77)	< 0.001	26.24(4.38)	25.03(3.96)	< 0.001	Women: < 0.001 Men: < 0.001
Heart rate, mean (SD)	88 (21.30)	79.44(20.07)	0.002	82.90(19.12)	76.71(18.90)	< 0.001	79.34(21.94)	76.52(21.90)	0.050	Women: 0.005 Men: 0.025
Previous MI, n (%)	2 (3.28)	46 (9.45)	0.108	15 (7.08)	146 (13.98)	0.006	37 (10.34)	90 (13.93)	0.101	Women: 0.072 Men: 0.032
Previous CABG, n (%)	1 (1.64)	11 (2.26)	0.754	6 (2.83)	33 (3.16)	0.798	9 (2.51)	32 (4.96)	0.062	Women: 0.062 Men: 0.872
C Heart failure, n (%)	2 (3.28)	10 (2.05)	0.538	11 (5.24)	23 (2.21)	0.014	23 (6.39)	25 (3.87)	0.072	Women: 0.169 Men: 0.753
Previous PCI, n (%)	2 (3.28)	26 (5.34)	0.491	8 (3.77)	86 (8.25)	0.024	18 (5.01)	41 (6.36)	0.386	Women: 0.085 Men: 0.706
Previous stroke, n (%)	1 (1.64)	6 (1.23)	0.789	15 (7.08)	40 (3.84)	0.036	33 (9.19)	66 (10.19)	0.612	Women: 0.113 Men: < 0.001
Previous angina, n (%)	10 (16.39)	65 (13.35)	0.514	61 (28.77)	157 (15.07)	< 0.001	86 (23.96)	95 (14.71)	< 0.001	Women: 0.249 Men: 0.125
Chest pain, n (%)	61 (100.0)	485 (100.0)	-	211 (100.0)	1039 (99.81)	0.524	353 (99.72)	642 (99.84)	0.668	Women: 0.681 Men: 0.636
Killip class, n (%) Class 1 Class 2 Class 3 Class 4	56 (91.80) 1 (1.64) 0 4 (6.56)	446 (91.58) 14 (2.87) 2 (0.41) 25 (5.13)	0.859	193 (91.04) 9 (4.25) 0 10 (4.72)	931 (89.18) 50 (4.49) 4 (0.38) 59 (5.65)	0.736	319 (88.12) 18 (4.97) 9 (2.49) 16 (4.42)	561 (86.44) 43 (6.63) 10 (1.54) 35 (5.39)	0.445	Women: 0.185 Men: 0.008
Current Smoking, n (%)	8 (3.11)	319 (65.50)	< 0.001	35 (16.51)	671 (64.27)	< 0.001	40 (11.05)	297 (45.69)	< 0.001	Women: 0.174 Men: < 0.001
Door-to-balloon time (min) (median (IQR))	103 (85-156)	90 (73-115)	0.071	96 (78-139)	94 (75-120)	< 0.001	105 (88-140)	99 (80-135)	0.206	Women: 0.542 Men: 0.007
Symptom to balloon time (min) (median (IQR))	325(240-450)	220(160-360)	< 0.001	330(195-465)	238(165-362)	< 0.001	295 (215-473)	282.5(200-430)	0.608	Women: 0.300 Men: < 0.001

BMI, body mass index; CABG, coronary artery bypass graft; PCI, Percutaneous coronary intervention *Chi-square/ Mann–Whitney U test for difference between men and women ** one-way ANOVA for difference between three age group according to men and women *P*<0.05 is statistically significant.

**Figure 1:**
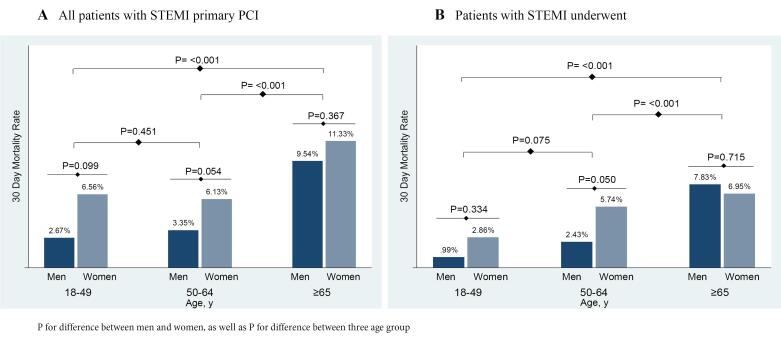
Unadjusted 30 day mortality rate according to age and sex

 In this study, differences were observed in various factors such as diabetes, heart rate, previous MI, previous stroke, Killip class, current smoking, door-to-balloon time, and symptom to balloon time, specifically within male age groups (*P* < 0.05). Notably, with the exception of the age group ≤ 65 with primary PCI, the 30-day death rate for all causes in both overall and primary PCI was found to be higher in women than in men across all three age groups, although the difference was not statistically significant. Furthermore, the mortality rate was observed to increase with age, with a considerable difference noted in the death rate between the young and middle-aged elderly (Figure 1). Additionally, significant disparities were identified in the administration of Aspirin within the middle-aged group 24 hours after hospitalization and at discharge. Moreover, a notable difference was observed between men and women in the administration of Clopidogrel, but only at the time of discharge. It is worth mentioning that no gender difference was noted in other treatment modalities. As age increased, there was a decrease in the proportion of individuals undergoing primary PCI, with 62% of men and 57% of women undergoing the procedure in the young age group, compared to 51% of both men and women in the older age group. Aspirin, Eptifibatide, primary PCI in men 24 hours after hospitalization, and Aspirin, Beta-blockers, Clopidogrel, and Statins at discharge show significant differences in age groups (Table 3). Predictors of 30-day death for all causes are presented in Table 4. A higher risk of 30-day death was reported for women (aged 50-64) than for men (adjusted HR: 2.76, 95% CI 1.09-7.0, *P* = 0.032). Additionally, the increased risk of the investigated outcome has a significant relationship with MI, PCABG, and Diabetes in the middle-aged. Younger individuals had a higher HR for mortality due to CHF compared to the elderly (Adjusted HR for young: 2.55, 95% CI 1.07-6.08, *P* = 0.035 VS Adjusted HR for older: 1.63, 95% CI 1.06-2.72, *P* = 0.046).

**Table 3:** Patient treatment and medications within 24 hours from onset and discharge

**Variable**	**18-49 (n=548)**			**50-64 (n=1256)**			**≥65 (n=1012)**		P*	***P*** for age**
	**Women (61)**	**Men (487)**	P*	**Women (212)**	**Men (1044)**	P*	**Women (362)**	**Men(650)**		
Medications within 24 hours from onset (n (%)) Aspirin Beta blockers Clopidogrel Eptifibatide	61 (100.0) 59 (96.72) 60 (98.36) 25 (40.98)	483 (99.18) 445 (91.35) 473 (97.13) 215 (44.15)	0.477 0.148 0.577 0.639	210 (99.06) 200 (94.34) 208 (98.11) 79 (37.26)	1043 (99.90) 943 (90.33) 1010 (96.74) 446 (42.72)	0.021 0.063 0.288 0.142	360 (99.45) 317 (87.55) 358 (98.90) 125 (34.53)	650 (100.0) 564 (86.77) 631 (97.08) 236 (36.31)	0.058 0.716 0.063 0.572	Women: 0.686 Men: 0.008 Women: 0. 006 Men: 0.21 Women: 0.738 Men: 0.891 Women: 0.565 Men: 0.010
Treatments (n (%)) Primary PCI Urgent CABG	35 (57.38) 0	303 (62.22) 0	0.464	122 (57.55) 0	617 (59.10) 3 (0.29)	0.675 0.434	187 (51.66) 1 (0.28)	332 (51.08) 4 (0.62)	0.859 0.460	Women: 0.342 Men: 0.000 Women: 0.685 Men: 0.185
Medications at discharge (n (%)) Aspirin Beta blockers Clopidogrel ACE inhibitors/ARB Statins	56 (91.80) 51 (83.61) 55 (90.16) 39 (63.93) 55 (90.16)	469 (96.30) 382 (78.44) 458 (94.05) 355 (72.9) 460 (95.24)	0.098 0.350 0.243 0.124 0.096	193 (91.04) 166 (78.30) 189 (89.15) 149 (70.30) 191 (90.09	998(95.59) 827 (79.21) 973 (93.20) 764 (73.18) 976 (93.76)	0.006 0.766 0.041 0.067 0.055	317 (87.57) 268 (74.03) 312 (86.19) 253 (69.89) 310 (85.87)	582 (89.54) 481 (74.0) 566 (87.08) 447 (68.79) 575 (88.46)	0.340 0.991 0.689 0.561 0.232	Women: 0.340 Men: 0.000 Women: 0.192 Men: 0.037 Women: 0.473 Men: 0.000 Women: 0.348 Men:0.027 Women: 0.274 Men: 0.000

*Chi-square for difference between men and women ** One-way ANOVA for difference between three age group according to men and women *P*<0.05 is statistically significant.

**Table 4:** Crude and adjusted hazard ratio of 30 days all causes mortality in STEMI patients

**Variable**	**18-49 (n=17)**			**50-64 (n=48)**			**≥65 (n=103)**		
	**HR**	**Confidence interval**	***P*** value*	**HR**	**Confidence interval**	***P*** value*	**HR**	**Confidence interval**	***P*** value*
Women Crude HR Adjusted HR	2.35 2.06	0.49-11.32 0.37-11.45	0.286 0.409	2.80 2.76	1.23-6.32 1.09-7.0	0.014 0.032	0.72 0.78	0.36-1.45 0.37-1.62	0.365 0.507
MI HR crude HR adjusted	NR NR			1.86 1.98	1.03-3.35 1.09-3.60	0.040 0.025	1.50 1.37	0.91-2.48 0.79-2.37	0.114 0.261
CHF Crude HR Adjusted HR	2.56 2.55	1.13-5.78 1.07-6.08	0.024 0.035	1.50 1.39	0.75-3.0 0.64-3.0	0.252 0.404	1.59 1.63	0.96-2.65 0.97-2.72	0.073 0.064
PPCI Crude HR Adjusted HR	2.31 1.94	0.29-18.45 0.22-17.08	0.430 0.551	1.09 1.37	0.26-4.62 0.32-5.93	0.908 0.673	1.93 2.27	0.685.43 0.80-6.48	0.213 0.125
PCABG Crude HR Adjusted HR	NR NR			4.42 4.83	1.32-14.76 1.42-16.40	0.016 0.012	NR NR		
Current smoking Crude HR Adjusted HR	0.84 0.81	0.22-3.13 0.19-3.41	0.796 0.778	0.83 0.94	0.32-1.82 0.41-2.16	0.649 0.882	0.85 0.79	0.43-1.67 0.39-1.62	0.636 0.523
Diabetes Crude HR Adjusted HR	1.66 1.81	0.34-7.97 0.33-9.87	0.529 0.493	2.61 3.19	1.18-5.74 1.34-7.61	0.017 0.009	0.74 0.80	0.34-1.61 0.35-1.83	0.453 0.595
Anterior Crude HR Adjusted HR	3.70 1.87	1.34-10.18 0.41-8.45	0.011 0.416	1.84 1.74	0.67-5.08 0.64-4.77	0.238 0.279	1.09 1.01	0.36-3.26 0.285-3.60	0.878 0.984

HR, hazard ratio; MI, Myocardial infarction; CHF, congestive heart failure; PCABG, previous coronary artery bypass graft; PPCI, previous percutaneous coronary intervention; NR, not run because of small numbers Adjusted for BMI, Diabetes, Hypertension and Dyslipidemia * Cox proportional hazards model for determine predictors of 30-day mortality *P*<0.05 is statistically significant.

## Discussion

 This study investigated the differences in the epidemiology, treatment, and mortality of STEMI patients based on age and sex in the Imam Ali Cardiology Center of Kermanshah, Iran registry. Our study revealed that young individuals had a higher average BMI, triglyceride levels, LDL levels, and current smoking rates compared to middle-aged and older individuals. Older individuals exhibited a higher prevalence of high blood pressure, diabetes, dyslipidemia, congestive heart failure (CHF), previous stroke, previous angina, and had a longer mean door-to-balloon time and symptom-to-balloon time. Across all three age groups, women demonstrated a higher prevalence of hypertension, diabetes, dyslipidemia, and elevated heart rate compared to men. Moreover, middle-aged and elderly women had higher rates of previous angina and BMI compared to men. Additionally, the median time from symptom onset to balloon inflation was shorter in men aged < 50 and 50-64 years compared to women. A higher ratio of current smoking and BMI in the American Heart Association’s (AHA)^17^ and Anna E. Bortnick^18^ studies in the age group of less than 45 years in America and the Pin Pin Pek study^19^ in Singapore in the age group of less than 65 years compared to older people has been shown, which is consistent with our study. In our study, the high prevalence of current smoking, BMI, TG, high LDL, and low HDL in the younger age group reflects bad habits and lifestyles in this group. In a cohort study in China, people aged 45 or less significantly had high LDL and low HDL compared to those over 45.^20^ Another study in Iran showed that young patients ( ≤ 35) had high LDL.^21^ Additionally, several epidemiological studies indicated that low HDL cholesterol is frequent in people aged 18–44 years, particularly in smokers.^22,23^ This shows the importance of intervention in risk factors and education in this age group. The use of beta-blockers has been shown to reduce the mortality rate in patients who have suffered from a myocardial infarction (MI), particularly in the elderly. Studies have also demonstrated that initial treatment with intravenous β-blockers can decrease mortality in patients with ST-segment elevation myocardial infarction (STEMI), making the use of β-blockers particularly beneficial for elderly patients.^24,25^ In the current study, over 89% of STEMI patients received β-blockers within the first 24 hours after admission. There was a noticeable difference in the percentage of young women who received β-blockers compared to older age groups within the first 24 hours after admission. Our study revealed that the 30-day mortality rate in STEMI patients who underwent primary percutaneous coronary intervention (PCI) ranged from 1.18% in the age group under 49 years to 7.51% in the age group of 65 years and older. The Pin Pin Pek study also noted that the one-year mortality rate in elderly STEMI patients undergoing primary PCI treatment increases from 20% at the age of 65 to 60% at the age of 85.^19^ The GUSTO-I trial observed a 10-fold increase in mortality in elderly STEMI patients compared to patients under 65 years of age.^26^ In the APEX-AMI trial, the 90-day mortality rate increased from 5.2% in the age group below 65 to 21.1% in the age group above 75. After adjusting for confounding factors such as baseline characteristics and clinical profile differences, age was reported as an independent predictor for 90-day attenuation in STEMI patients with primary PCI.^27^ The high death rate in the elderly can be attributed to the frequency of electrical and mechanical catastrophes.^28^ Age-related mechanical catastrophes can be justified by changes in heart physiology or reduced vascular compliance, as well as ventricular hypertrophy in the elderly. In a large American study of 25,353 STEMI patients, the mortality rate was higher in women compared to men, at 10.2% versus 5.5%, respectively.^29,30^ This higher rate in women was only evident in deaths within 24 hours after admission.^10^ A meta-analysis of STEMI patients treated with primary PCI indicated that all-cause mortality was higher in women, but this difference was likely confounded by baseline cardiovascular risk factors and medical records.^31^ The sex difference in mortality in the Bortnick study was observed only in the 45-64 age group, although this difference was not significant after adjusting for demographic and clinical factors.^18^ Another study in America, after extensive adjustment for co-morbidity, did not show higher 30-day hospital mortality in middle-aged and elderly women. However, other factors such as atypical presentation and delay in treatment can affect the outcomes of STEMI in women.^32^ Another study in 2006 demonstrated when the angiographic severity of coronary artery disease is matched with baseline characteristics for 30-day mortality, there is no significant difference between sex and mortality, including the interaction between age and gender on mortality.^14^ In our study, women exhibited a higher mortality rate STEMI patient in the absence of primary PCI. Only men aged 65 or older had a higher mortality rate in patients who underwent primary PCI. However, the difference between men and women in young and old patients is not statistically significant. The middle age group is borderline; the lack of statistical significance may be attributed to the lower proportion of women compared to men (22.55% vs. 77.45%) and the differences in the risk factor profile of women in the present registry. Part of the disparity between men and women could be attributed to inequality and treatment delays. Longer door-to-balloon time, less evidence of treatment, such as aspirin, β-blockers, and reperfusion therapies, and lower primary PCI, are among the contributing factors to the higher death rate in women compared to men.^31,33^ However, our study did not find any difference in treatment between men and women. Other factors responsible for the varying outcomes between men and women with STEMI may be related to vascular biological factors, such as smaller vessel size, less collateral flow, and increased vascular stiffness in women.^34,35^ Men and women with STEMI exhibit different cardiovascular risk factors and factors that influence mortality based on age. One limitation of this study is the inability to ascertain the socio-economic status, access to, and delay in the treatment of patients. Although the risk of death was adjusted for clinical risk factors in this study, residual confounding may still persist. Additionally, the data for this study were obtained from a registry center; while this center is the sole referral center for STEMI patients in the province, it may be susceptible to selection bias.

## Conclusion

 Women had a higher mortality rate than men. However, this difference is not significant, possibly due to the lower proportion of women in this registry and the difference in the profile of cardiac risk factors. Elderly patients receive less primary PCI and have a longer door-to-balloon time and symptoms-to-balloon time than middle-aged and young patients. The current study emphasizes intervention and education for young age groups and women to improve lifestyle, clinical, and epidemiological risk factors.

## Acknowledgments

 The authors thank the Kermanshah University of Medical Sciences for funding this project. We wish to thank all the Imam-Ali hospital staff, especially the Cardiovascular Research Center, Dr. Hossein Siabani, Ms. Leila Zamzam, Mrs. Hanyeh Charejo and Ms. Elaheh Mohammadi for data gathering; without their contribution, this work would not have been accomplished.

## Competing Interests

 The authors declare that there were no conflicts of interest. In addition, the authors have no financial gain related to any aspect of the study.

## Ethical Approval

 The Research Ethics Committee at KUMS approved the study protocol (Ethics No. KUMS.REC.1395.252). Also, patients were informed about participating in the registry and signed the consent form. Patient data were kept confidential with the access limited to two of researchers and the quality control physician.

## Funding

 This study was supported by Kermanshah University of Medical Sciences.
